# Malignancy in neuromuscular patients on chronic IVIG

**DOI:** 10.3389/fneur.2025.1571160

**Published:** 2025-06-24

**Authors:** Mohamed Khateb, Elian Bahous, Mai Abu Zant, Shahar Shelly

**Affiliations:** ^1^Department of Neurology, Rambam Medical Center, Haifa, Israel; ^2^Rappaport Faculty of Medicine, Technion-Israel Institute of Technology, Haifa, Israel; ^3^Department of Neurology, Mayo Clinic, Rochester, MN, United States

**Keywords:** Myasthenia Gravis, CIDP, IVIG, cancer, autoimmune disorders

## Abstract

**Background:**

Intravenous immunoglobulin (IVIG) has been widely used to treat immune-mediated neuromuscular disorders. The relationship between IVIG and cancer is unclear. Preclinical studies have suggested that IVIG may influence cellular mechanisms pertinent to cancer development. This hypothesis is supported by clinical evidence, predominantly through case reports, although these findings are limited in scope and methodological rigor.

**Methods:**

A retrospective review was conducted on patients receiving chronic IVIG treatment for Myasthenia Gravis (MG) and chronic immune demyelinating polyradiculoneuropathy (CIDP), at our tertiary medical center between 2000 and 2023.

**Results:**

We identified 436 patients with MG and 102 patients with CIDP. Patients were divided into IVIG and non-IVIG treated in each of the disease groups. Seventy-five and 64 patients received IVIG in MG and CIDP groups, respectively. Cancer incidence was counted if it appeared at least 1 year after the diagnosis of MG or CIDP. In the MG group, cancer incidence was 12 / 75 (16%) among IVIG-treated patients compared to 87/350 (25%) in the MG-non-IVIG group (*p* = 0.09). Excluding mild MG cases, who were treated only by pyridostigmine, cancer incidence in the MG-non-IVIG group increased to 72/250 (28.8%), significantly higher than in the IVIG-MG group, *p* = 0.01. For CIDP patients receiving IVIG, incidence of cancer was 6/59 (10%), while in the non-IVIG-treated CIDP group, it was 9/34 (26%) (*p* = 0.03). Analyzing all IVIG-treated patients together (of both disease groups) showed a negative correlation between “time on IVIG treatment” and cancer (*p* = 0.001). Using logistic regression, we observed a negative coefficient for IVIG exposure (−0.03, *p* = 0.004), indicating that an increase in time from diagnosis is associated with a decreased likelihood of developing cancer in the IVIG-treated group.

**Conclusion:**

Chronic IVIG therapy may be associated with a reduced incidence of cancer, particularly among patients with CIDP. Additionally, we found a negative correlation between duration of IVIG treatment and cancer incidence, suggesting a potential protective effect of long-term IVIG exposure. To our knowledge, this is the first study to explore the relationship between sustained IVIG therapy and long-term cancer risk in neuromuscular autoimmune diseases.

## Introduction

Intravenous immunoglobulins (IVIG), a relatively safe formulation of over 95% IgG immunoglobulins derived from thousands of healthy donors’ plasma (1, 2). IVIG has shown strong anti-inflammatory and immunomodulatory effects in diseases like immunodeficiencies, autoimmune diseases, and infections. However, due to production challenges and high costs, there is a global shift toward novel biological treatments (3–6). Investigations into the efficacy of IVIG have been extensive, particularly the impact on autoimmune disorders, especially Myasthenia Gravis (MG), Chronic Inflammatory Demyelinating Polyneuropathy (CIDP) and other immune-mediated neuropathies including multifocal motor neuropathy (MMN), anti-MAG neuropathy, Neurofascin-155 seropositive immune-mediated neuropathy, chronic immune sensory polyradiculopathy (CISP), and more (7–10). Despite this, the direct impact of IVIG on neoplasms has not been thoroughly explored in past research, leaving its effects largely unknown.

Previous preclinical studies demonstrated IVIG-induced effects on IL-12, NK activity, and MMP-9 all directly linked to anti-neoplastic response. Notably, IVIG reduced metastasis of melanoma and sarcoma in C57BL/6 J and severe combined immunodeficient (SCID) mice that were injected with either melanoma or sarcoma cells and then treated with IVIG (11–13). The mechanism of this effect included the enhancement of IL-12 secretion and increased NK activity. In the cellular level, key mechanisms related to tumoral surveillance were suggested. IVIG affects cellular proliferation by arresting G1 as a result of p21/WAF-1 up-regulation, elevation of p53 expression, NF-kB and anti-BAFF activity (11, 14–17). Activating the FcgR was suggested also as an anti-tumoral mechanism (13, 18). Additional mechanism is related to recognizing oligosaccharides expressed on tumor cells. IVIG contains a wide spectrum of specificities presented in normal plasma including natural antibodies that recognize these oligosaccharides (19). Furthermore, IVIG was found to comprise anti-angiogenic activity *in vitro* and *in vivo* thus possibly inhibiting metastasis (19, 20).

Clinically, data mainly from case reports tried to correlate IVIG with decreased tumoral activity (21–25). More specifically, IVIG was associated with inducing tumor stability and prolonged survival time in a patient with metastatic melanoma. In another patient with malignant peripheral nerve sheath tumor, the disease course was longer and more indolent than expected. Interestingly, a regression of a Kaposi’s Sarcoma was observed with an HIV patient. Furthermore, beneficial effects of IVIG were suggested in lymphoproliferative disorders (21, 26, 27). Nevertheless, it is not clear whether these beneficial effects were achieved because of treating complications like the secondary immunodeficiency state in these diseases or direct anti-lymphoproliferative effects or effects against oncogenic pathogens like EBV, CMV and more (28, 29).

To our knowledge, no controlled large studies were performed to further investigate or support this anti-neoplastic trait of IVIG. IVIG contains a wide spectrum of proteins participating in cancer response pathways (e.g., anti-DNA, β2glycoprotein-I, Fas, Arg-Gly-Asp (RGD) motif, B cell-activating factor of the tumor necrosis factor (TNF) and more) (11, 30). Evidence regarding the impact of IVIG on cancer remain scattered and inconclusive, primarily due to a lack of controlled clinical trials. In our study, we set out to examine the influence of IVIG on cancer occurrence within two distinct autoimmune patient groups: MG and CIDP, exploiting the fact that some of these patients are on maintenance IVIG for disease control. We compared the cancer incidence in patients undergoing chronic IVIG therapy to those treated with other immunosuppressive methods. By investigating two different autoimmune diseases, our goal is to bolster the reliability of our findings and determine whether IVIG has any potential anti-neoplastic effect immune-mediated disorders of the peripheral nervous system.

## Materials and methods

### Study design ethical considerations, and study cohort

This retrospective cohort study was conducted after obtaining approval from our institutional IRB Helsinki Committee. The study was exempted from the informed consent procedure due to its retrospective nature, based on existing data in the medical records.

The files of patients with confirmed diagnoses of Myasthenia Gravis and CIDP disorders who received medical attention at a single tertiary medical facility from January 1, 2000, to December 31, 2023, were screened. The first group included patients with MG diagnosis, that was based on the combination of both a clinical picture of MG and a supportive electrodiagnostic (EDX) testing (31, 32) or serology [Ach receptor verified by cell-based-arrays/CBAs or muscle-specific tyrosine-kinse (MuSK)]. Exclusion criteria were elaborated in our previous papers (33, 34). Notably, we reassured the absence of any alternative diagnosis explaining the clinical presentation, the laboratory, or the electrodiagnostic findings. Two-Hertz motor RNS with a train of 4 stimuli was utilized to assess for a postsynaptic neuromuscular defect. RNS was deemed confirmatory when a physiologic pattern of decrement of >10% was seen in the compound muscle action potential at baseline or up to 3 min post-1-min exercise in 2 or more motor nerves without alternative explanation such as myopathy, neuropathy, or motor neuron disease. Stimulatory single-fiber EMG (SFEMG) was also performed in suspected patients. SFEMG positivity was determined utilizing quality and cutoff guidelines previously published (31, 32). A double seronegative serologic status was not considered an exclusion criterion as part of myasthenic patients are known to be seronegative.

Regarding the CIDP cohort, all patients have had a clinical presentation of chronic (≥8 weeks since symptoms’ onset) and progressive or relapsing polyneuropathy. All patients had nerve conduction studies that were ‘strongly supportive of demyelination’ according to the European Federation of Neurological Societies (EFNS) Task Force (2021) (35). The clinical presentation was divided into typical CIDP or atypical CIDP variant. The former relates to a typical presentation of progressive or relapsing, symmetric, proximal and distal muscle weakness at upper and lower limbs, and sensory involvement of at least two limbs with reduced or absent tendon reflexes at all limbs. Atypical CIDP variants include distal CIDP/distal acquired demyelinating symmetric neuropathy (DADS) variant, multifocal CIDP (MADSAM), sensory-predominant CIDP variant, motor CIDP variant, and focal CIDP. All these variants were defined according to the EFNS Task Force (2021). In addition, an extended laboratory polyneuropathy workup was conducted for all patients to rule out other causes for neuropathy. This workup included HBA1C, glucose fasting, 2-h-glucose-tolerance-test, Thyroid stimulating hormone (TSH), B12, folic acid, serum protein electrophoresis with immunofixation, kappa and lambda free light chains, Ana screening, and collagenogram, serological testing for HIV, HBV, HCV, Syphilis and west nile virus. Patients suspected of a genetic etiology (especially with positive family history) or with a history of chronic alcohol consumption were not included.

### Data compilation

We systematically collected demographic and clinical information from the medical records of subjects meeting the eligibility criteria. The occurrence of cancer was determined through cross-referencing with the hospital’s archives. Each case underwent a thorough evaluation (M.K. and S.S.). During the study period, we identified 436 patients diagnosed with MG, excluding 11 due to insufficient follow-up (lack of follow-up data within the last 5 years). Of the 425 eligible patients, 350 did not receive Intravenous Immunoglobulin (IVIG) therapy, while 75 underwent maintenance treatment with IVIG. Furthermore, 102 patients were diagnosed with Chronic Inflammatory Demyelinating Polyneuropathy (CIDP), based on the criteria established by the European Federation of Neurological Societies (EFNS) Task Force (2021), as previously elaborated (35). Among these, 64 patients received chronic maintenance treatment with IVIG, whereas 38 were managed without IVIG therapy. Cases in which IVIG was administered solely for acute exacerbations were excluded from analysis. This methodology ensures a comprehensive assessment of the therapeutic interventions and their associations with clinical outcomes in patients with MG and CIDP disorders within the study population.

### Cancer association

We reviewed cases across the two disease cohorts (MG and CIDP) to affirm the presence of malignancies and accurately categorize them based on the affected organ. The analysis was conducted using the MDClone platform (MDClone Ltd., Beer Sheva, Israel), a tool that integrates seamlessly with our electronic medical records (EMR) system. For the classification of cancer, we considered various parameters including the date of cancer diagnosis, the patient’s age at diagnosis, the specific cancer subtype as identified in pathology and oncology reports, and the time interval between the diagnosis of the autoimmune disease (MG or CIDP) and the cancer diagnosis. The date of autoimmune disease diagnosis was established as time zero for our analysis. This zero point was chosen as the disease diagnosis time, rather than the IVIG initiation time, in order to keep homogeneity between the groups with and without IVIG within each of the disease groups. We employed a rigorous validation process for our data, incorporating continuous oncological clinical monitoring and pathological confirmation for cases. The identification of malignancies was based on the anatomical location of the primary tumor, excluding conditions such as premalignant skin changes, prostate hyperplasia, gastrointestinal polyps, and premalignant hematologic disorders, among others. In instances of multiple cancer diagnoses within the study period, priority was given to the cancer diagnosis most temporally proximate to the autoimmune diagnosis for the calculation of cancer associations.

### Objectives and statistical analysis

**Figure 1:**
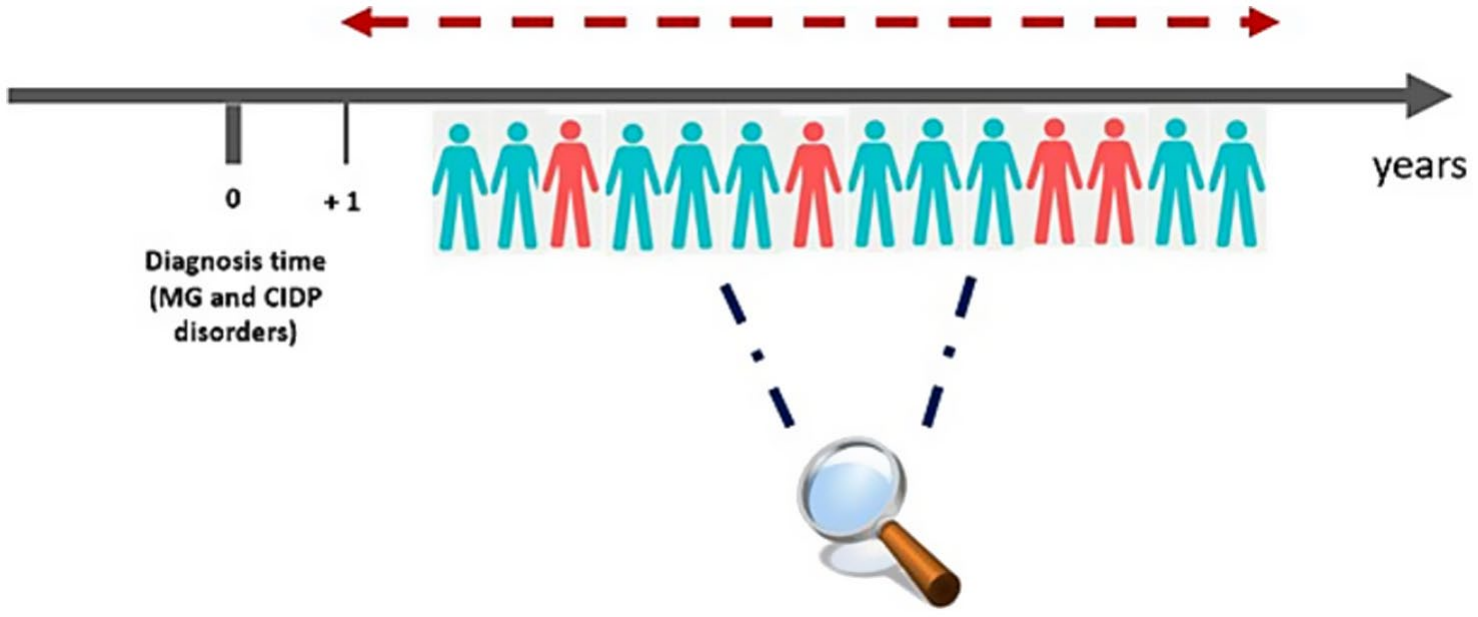
Schematic view of the study. Patients were investigated regarding the incidence of cancer in each of the disease groups (MG and CIDP). The date of autoimmune disease diagnosis was established as time zero for the purpose of our analysis. The dashed red arrow represents the time interval of cancer investigation (after at least 1 year of zero time). Red-colored Individuals represent patients with a cancer diagnosis while those who are green colored did not develop any kind of cancer.

The primary objective was the *change* in cancer incidence in each of the disease groups, CIDP and MG patients, who were treated with chronic IVIG versus patients treated with no IVIG (Figure 1). Cancers were counted if diagnosed after the autoimmune diagnosis by at least one year. Within the IVIG groups (IVIG-MG and IVIG-CIDP), we verified that cancers’ diagnosis was established after the IVIG initiation time, otherwise they were not counted. Cancers diagnosed within the same year as autoimmune disorder were excluded from the analysis. This comparison was performed using the Pearson Chi-square test. Descriptive statistics used to summarize the characteristics of the study population. A two-tailed *p*-value < 0.05 was considered statistically significant. For the correlation of IVIG with time, Mann–Whitney U test was used to compare the medians.

## Results

### Patient characteristics and clinical features in each group

For the MG cohort, we identified 436 patients with MG diagnosis. Eleven patients were excluded due to lack of adequate follow up time. Total 425 patients included in the analysis (IVIG group = 75; No-IVIG = 350), median age at symptoms onset was 52 (14–89 years) at the IVIG group and 60 (5–93 years) for the non-IVIG group (Table 1, *p* = 0.03). Males versus female’s ratio was similar between the IVIG and the non-IVIG groups; 41.33% (31/75) and 46.6% (163/350) for the males, and 58.7% (44/75) and 53.4% (187/350) for the females, *p* = 0.41. MG symptoms at disease presentation were recorded as ocular (60%, 260/436), or generalized (40%, 176/436) onsets. Overall median follow-up time was 3 (range: 1–34 years) with an average of 5.2 years for both groups. For the CIDP cohort using EFNS 2021 criteria, we identified 102 patients (IVIG group = 64; non IVIG group = 38), median age at symptoms onset was 56 (range: 19–88 years) and 59 (range: 25–80) at the IVIG and the non-IVIG groups respectively, *p* = 0.49. Males to female’s ratio was similar between the two groups (Table 1, *p* = 0.94). The largest group of this CIDP cohort (43.1%, 44/102) were typical CIDP patients, while the remaining 58/102 were composed of atypical CIDP presentation. Of the 58 atypical patients, the leading variant was sensory-predominant (24/102), followed by the distal acquired demyelinating symmetric neuropathy (DADS) variant (16/102), multifocal/MADSAM (9/102), pure ataxic (5/102), pure motor (3/102) and focal onset of one limb (1/102). The median time for IVIG treatment in the IVIG-MG and IVIG-CIDP groups was 1.75 (0.5–20) and 1.5 (0.25–12) years, respectively.

**Table 1:** Demographic features, treatments and cancer incidence in each of the disease groups.

Variable	MG (IVIG) *n* = 75	MG (non IVIG) *n* = 350	*p* value	CIDP (IVIG) *n* = 64	CIDP (non-IVIG) *n* = 38	*p* value
Age at diagnosis in years (median, range)	52 (14–89)	60 (5–93)	0.03	56 (19–88)	59 (25–80)	0.49
Females ratio	58.7% (44/75)	53.4% (187/350)	0.4	30% (19/64)	29% (11/38)	0.94
Cancer cases^*^	16% (12/75)	25% (87/350)	0.09	10% (6/59)	26% (9/34)	0.03
Age at cancer diagnosis in years (median and range)	70 (38–84)	72 (19–94)	0.285	67.5 (50–78)	72 (46–80)	0.61
IVIG Treatment time in years (median)	1.75 (0.5–20)	NA	NA	1.5 (0.25–12)	NA	NA
Dose IVIG /month (gr)	35.125 (20–60)	NA	NA	34.81 (20–60)	NA	NA
Steroids (number of patients and ratio from 100%)	59 (78.7%)	208 (59.4%)	0.0017	25 (39.1%)	12 (31.6%)	0.452
No steroids (number of patients and ratio from 100%)	16 (21.3%)	142 (40.6%)	0.0017	39 (60.9%)	26 (64.8%)	0.452
Non-steroidal immunosuppressants; Azathioprine, Mycophenolate Mofetil, Methotrexate, Cyclophosphamide (number of patients and ratio from 100%)	45 (60%)	157 (44.9%)	0.017	15 (23.44%)	11 (28.94%)	0.541
No Non-steroidal immunosuppressants (number of patients and ratio from 100%)	30 (40%)	193 (55.1%)	0.018	49 (76.56%)	27 (71.06%)	0.549
Smokers (number of patients and ratio from 100%)	9 (12%)	51 (14.6%)	0.56	13 (20.31%)	8 (21.05%)	0.93

^*^All extrathymic cancer cases counted after initiation of IVIG or other standard treatment.

### IVIG and cancer associations

**Figure 2:**
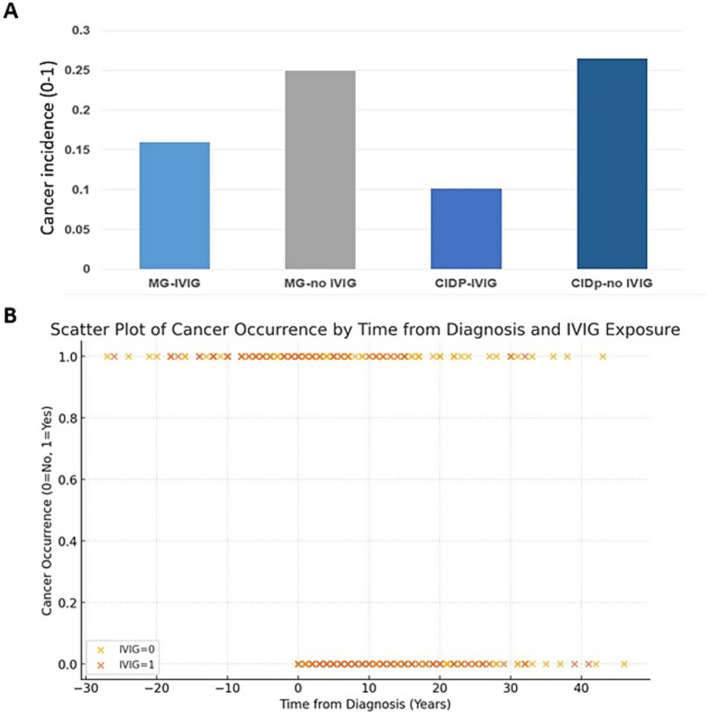
The effect of IVIG on cancer incidence in each of the disease groups. **(A)** Cancer incidence is presented for each of the disease groups with or without chronic IVIG treatment. The X-axis represents the patient’s group while the Y-axis enumerates cancer incidence during the time interval after MG/CIDP diagnosis. In both disorders, the IVIG group had lower cancer incidence in the post-diagnosis time compared with the non-IVIG groups (*p* = 0.09 and *p* = 0.03 for MG and CIDP, respectively). **(B)** Scatter Plot of Cancer Occurrence by Time from Diagnosis and IVIG Exposure. This scatter plot illustrates the relationship between the time from diagnosis (in years) and the occurrence of cancer, with data points representing individual patients in both of the disease groups MG and CIDP, combined. The x-axis shows the time from diagnosis, while the y-axis indicates cancer occurrence (0 = No cancer, 1 = Cancer). Patients are categorized by their IVIG exposure status: those who received IVIG (IVIG = 1) and those who did not (IVIG = 0). Each point is color-coded based on IVIG status.

We compared cancer incidence within groups between cases who were exposed to IVIG vs. non-exposed in each of the diseases (Table 1; Figure 2). In the IVIG-MG group, total number of cancers diagnosed after the zero time (which refers to the diagnosis time of MG) was 12/75 (16%) versus MG-non-IVIG group with 87/350 (25%), *p* = 0.09, OR = 1.5; CI:0.89–2.69. Since the average age of onset at the MG-IVIG group was younger than at the MG-non IVIG group, we mitigated this difference by considering cancer’s ratio in the time interval preceding the MG diagnosis that were younger. Cancer incidence was 13.33% (10/75) and 11.71% (41/350) at the MG-IVIG and MG-non IVIG groups, respectively (*p* > 0.05). This similarity regarding cancer incidence during the pre-diagnosis time interval between the two groups indicates that the increase in cancer incidence observed in the MG-non-IVIG group, after MG diagnosis, compared to the MG-IVIG group, is not related to differences in age of onset. Excluding the mild MG cases, who were treated only by Pyridostigmine without any immunosuppressant agents (100/436), cancer incidence in the MG-non-IVIG group increases to 72/250 (28.8%), which proves to be statistically significant compared to the cancer incidence in IVIG-MG group (12/75, 16%, *p* = 0.013).

**Figure 3:**
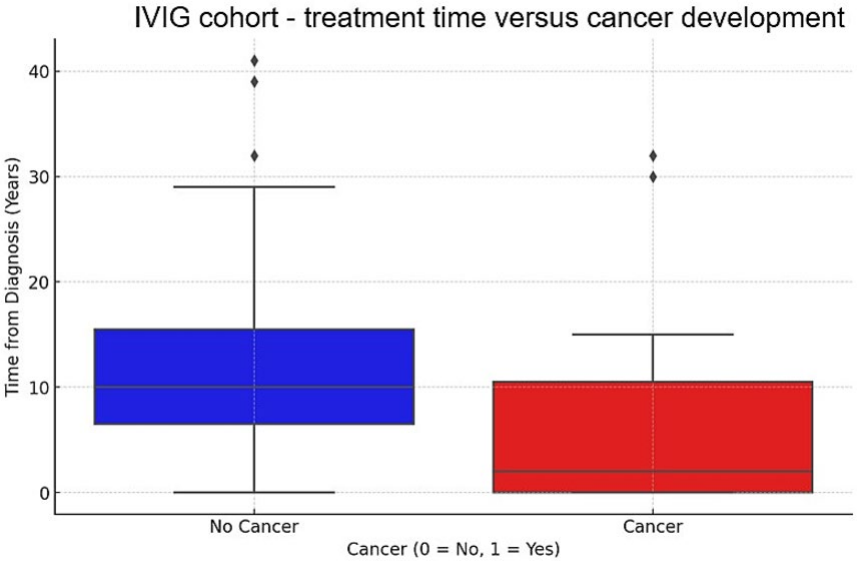
Box plot of time under IVIG treatment until cancer development or last follow up for patients who developed cancer vs. those who did not. This box plot compares the distribution of time under chronic IVIG treatment between patients who developed cancer (red) and those who did not (blue). The y-axis represents the time in years under IVIG treatment until cancer diagnosis or last follow up (if no cancer developed). The plot shows the median, quartiles, and outliers for each group. Patients who did not develop cancer (blue) generally received IVIG for a longer time (median around 10 years), while those who developed cancer (red) received IVIG treatment for shorter time durations (median around 2 years). Outliers are indicated by dots outside the whiskers.

CIDP-IVIG total number of cancers appearing after the diagnosis time of CIDP was 6/59 (10%) and 9/34 (24%), *p* = 0.03, OR = 3.18, CI: 1–6.6. Combining the two disease groups together, a tendency of reducing cancer incidence is observed *p* = 0.06 (Figure 3). The association is not statistically significant but it is close to significance. Risk ratio is 1.228 (CI = 0.97 to 1.55), indicating a higher cancer risk in non IVIG exposed group.

The Protective Effect of IVIG was analyzed using logistic regression analysis, suggested a negative coefficient for IVIG exposure (−0.03, *p* = 0.004), implying that as the time from diagnosis increases, the likelihood of developing cancer decreases in IVIG treated group (Figure 3). This suggests that IVIG exposure may reduce cancer risk. In MG patients, the average treatment time of IVIG in MG (75 patients) was 3.7 years (range: 0.5–20 years) and the average dose was 35.125 gr/month (range: 20–60 gr/month). The average treatment time for CIDP patients receiving IVIG (64 patients) was 2.84 years (range: 0.25–12) and the average dose per month was 34.81 gr (range: 20–60 gr/month). IVIG significantly affected gastrointestinal cancers (*p* = 0.0175, Table 2) and was nearly significant for dermatologic cancers (*p* = 0.11, Table 2).

**Table 2:** Cancer types and histology according to treatment with IVIG, combining the two disease groups.

Cancer type	IVIG (139)	Histological details	No IVIG (388)	Histological details	*p* value (between columns 1 and 3)
Gastrointestinal	1 (0.72%)	Pancreatic pseudo-papillary carcinoma (1)	21 (5.41%)	Colorectal adeno carcinoma (12), Gastric adeno carcinoma (2), Pancreatic head cancer (1), Pancreatic IPMN carcinoma (1), Pancreatic carcinoma with no further details (1), GIST of stomach (1), Squamous cell carcinoma of the esophagus (1), Hepatocellular carcinoma (1), Gallbladder adenocarcinoma (1).	0.0175
Dermatologic	3 (2.16%)	Squamous cell carcinoma (2), Basal cell carcinoma (1)	21 (5.41%)	Squamous cell carcinoma (14), Basal cell carcinoma (4), Metastatic melanoma (3).	0.114
Renal and urinary tract	3 (2.16%)	Papillary urothelial carcinoma (2), Renal cell carcinoma (1)	11 (2.84%)	Renal cell carcinoma (4), Papillary urothelial carcinoma (4), Prostate adenocarcinoma (3).	0.671
Hematologic	5 (3.6%)	Chronic lymphocytic leukemia (1), Acute myeloid leukemia (1), Follicular lymphoma (1), Multiple Myeloma (1), Hodgkin Lymphoma (1).	11 (2.84%)	Chronic lymphocytic leukemia (2), Multiple Myeloma (2), Acute myeloid leukemia (2), Diffuse large B cell lymphoma (1), Plasmacytoma (1), Chronic myeloid leukemia (1), Waldenstrom macroglobulinemia (1), MALT lymphoma (1)	0.654
Lung	3 (2.16%)	Adenocarcinoma (1), Small cell carcinoma (1), Squamous cell carcinoma (1).	13 (3.35%)	Squamous cell carcinoma (5), Adenocarcinoma (4), Non-small-cell carcinoma with no further details (3), Small cell carcinoma (1)	0.483
Breast	1 (0.72%)	Invasive ductal carcinoma (1)	7 (1.8%)	Invasive ductal carcinoma (5), Breast carcinoma with no further details (2)	0.37
Genital/pelvic	1 (0.72%)	Squamous cell carcinoma of uterine cervix (1)	7 (1.8%)	Endometrial carcinoma (3), Squamous cell carcinoma of uterine cervix (2), malignant seminoma (1), Embryonal testicular carcinoma (1)	0.37
Endocrine	1 (0.72%)	Lung carcinoid (1)	2 (0.52%)	Adrenocortical carcinoma (1), Thyroid papillary carcinoma (1).	0.784
Mouth and larynx	0 (0%)	N/A	1 (0.26%)	Squamous cell carcinoma (1)	N/A
Nose and sinuses	0 (0%)	N/A	2 (0.52%)	Squamous cell carcinoma (1), Maxillary sinus cancer with no further details (1)	N/A

## Discussion

In this single-center retrospective study, we evaluated 139 patients diagnosed with CIDP or MG who received maintenance IVIG and had long-term follow-up. We compared these individuals to similar disease control groups of MG and CIDP patients treated without IVIG. Our findings indicate a significantly lower incidence of cancer in the IVIG-treated groups, suggesting an association between chronic IVIG treatment and reduced cancer rates. This study is the first to explore the long-term effects of maintenance IVIG therapy on cancer incidence in patients with chronic autoimmune diseases of the peripheral nervous system. Prior research focused on the direct immunomodulating effect of IVIG and has not conclusively linked IVIG use to reduced cancer risk. Our results suggest potential anti-cancer effects of IVIG in neuromuscular autoimmune disorders.

Previous research, including our recently published studies, has shown increased cancer incidence in MG and other autoimmune disorders, linking these conditions to a higher cancer risk (33, 36–41). The exact cause behind this increase is debatable, with some suggesting mechanisms related to the chronic autoimmune state, other mechanisms related to the use of chronic immunosuppression (42–44) Interestingly, IVIG treatment exposure significantly decreases cancer incidence in the CIDP group. In the MG group, this effect became statistically significant only after excluding mild MG cases that were treated with pyridostigmine monotherapy. This result can be attributed to the severely compromised autoimmune condition in challenging MG cases, which further elevates the risk of cancer. Our study also explored the relationship between cancer incidence and time of IVIG treatment. We found that time on IVIG possibly relates to its protective effect against cancer (Figure 3). These results underscore IVIG’s potential anti-neoplastic benefits, previously hinted at in isolated reports but now supported by more robust data.

IVIG is thought to modulate cancer through its broad specificity and influence on various cellular mechanisms, offering both anti-tumoral and anti-metastatic effects. Its anti-tumor actions include halting cell proliferation by upregulating p21/WAF-1, enhancing p53 expression, and inhibiting BAFF and MMP9, alongside activating FcgR and recognizing tumor cell oligosaccharides through natural antibodies in the plasma (11, 13, 14, 17–19). For anti-metastasis, IVIG reduced melanoma and sarcoma spread in experimental models by boosting IL-12 and NK cell activity and suppressing MMP-9, also demonstrating anti-angiogenic properties that hinder metastasis development (19, 20).

Our study has several limitations. Its retrospective, single-center design may introduce biases from historical patient data. The perceived protective effect of IVIG against cancer might not be due to the absence of pro-oncogenic factors like immunomodulators, smoking, etc. Both groups had similar numbers of patients using steroids, immunosuppressants, and smokers in the CIDP disease group (Table 1). In the MG group, patients on chronic IVIG even received, on average, more treatments with steroids and immunosuppressant agents. Hence, external pro-oncogenic factors are unlikely to explain the observed differences in cancer incidences. In the MG-IVIG group, more patients were on prednisone and immunosuppressants compared to the MG without IVIG group, likely due to the presence of more severe cases. No direct head-to-head comparison was performed between IVIG and each of the other medications used in the non-IVIG groups. The younger average age of IVIG recipients in the MG cohort may affect cancer incidence rates, though we attempted to normalize this by pre-diagnosis incidence levels. Similar pre-diagnosis cancer incidence between MG groups suggests the increase in post-diagnosis cancer incidence among MG-IVIG patients is related to MG itself. Despite these limitations, the study’s strengths include control groups from the same center and detailed long-term follow-up, bolstering our findings’ validity.

Our study suggests that IVIG treatment may reduce cancer incidence in patients with MG and CIDP, with the most pronounced effect observed in those receiving long-term IVIG therapy. This reduction was statistically significant in CIDP and in MG patients with moderate to severe disease requiring immunotherapy. Although newer biologic agents are increasingly used for their targeted mechanisms, our findings support the continued use of IVIG as a core therapeutic option—particularly in patients with coexisting or suspected malignancy. These results provide new insight into the potential dual role of IVIG in both immunomodulation and cancer risk reduction in chronic autoimmune neuropathies.

## Data Availability

The raw data supporting the conclusions of this article will be made available by the authors, without undue reservation for authorized investigators.
